# Harm reduction in undergraduate and graduate medical education: a systematic scoping review

**DOI:** 10.1186/s12909-023-04931-9

**Published:** 2023-12-21

**Authors:** Kelsey R. Smith, Nina K. Shah, Abby L. Adamczyk, Lara C. Weinstein, Erin L. Kelly

**Affiliations:** 1 University of California San Diego School of Medicine, 9500 Gilman Dr, La Jolla, CA 92093 USA; 2 Department of Family and Community Medicine, Thomas Jefferson University, 1015 Walnut St, Curtis Building, Philadelphia, PA 19107 USA; 3 Sidney Kimmel Medical College, Thomas Jefferson University, 1025 Walnut St, #100, Philadelphia, PA 19107 USA; 4 Scott Memorial Library, Thomas Jefferson University, 1020 Walnut St, Philadelphia, PA 19107 USA; 5 Center for Social Medicine and Humanities, University of California Los Angeles, B7-435, Semel Institute, Los Angeles, CA 90095-1759 USA

**Keywords:** Harm reduction, Substance use, Opioid use disorder, Undergraduate medical education, Graduate medical education

## Abstract

**Background:**

Substance use increasingly contributes to early morbidity and mortality, which necessitates greater preparation of the healthcare workforce to mitigate its harm. The purpose of this systematic scoping review is to: 1) review published curricula on harm reduction for substance use implemented by undergraduate (UME) and graduate medical education (GME) in the United States and Canada, 2) develop a framework to describe a comprehensive approach to harm reduction medical education, and 3) propose additional content topics for future consideration.

**Methods:**

PubMed, Scopus, ERIC: Education Resources Information Center (Ovid), and MedEdPORTAL were searched. Studies included any English language curricula about harm reduction within UME or GME in the United States or Canada from 1993 until Nov 22, 2021. Two authors independently reviewed and screened records for data extraction. Data were analyzed on trainee population, curricula objectives, format, content, and evaluation.

**Results:**

Twenty-three articles describing 19 distinct educational programs across the United States were included in the final sample, most of which created their own curricula (*n* = 17). Data on educational content were categorized by content and approach. Most programs (85%) focused on introductory substance use knowledge and skills without an understanding of harm reduction principles. Based on our synthesis of the educational content in these curricula, we iteratively developed a Harm Reduction Educational Spectrum (HRES) framework to describe curricula and identified 17 discrete content topics grouped into 6 themes based on their reliance on harm reduction principles.

**Conclusions:**

Harm reduction is under-represented in published medical curricula. Because the drug supply market changes rapidly, the content of medical curricula may be quickly outmoded thus curricula that include foundational knowledge of harm reduction principles may be more enduring. Students should be grounded in harm reduction principles to develop the advanced skills necessary to reduce the physical harm associated with drugs while still simultaneously recognizing the possibility of patients’ ongoing substance use. We present the Harm Reduction Educational Spectrum as a new framework to guide future healthcare workforce development and to ultimately provide the highest-quality care for patients who use drugs.

**Supplementary Information:**

The online version contains supplementary material available at 10.1186/s12909-023-04931-9.

## Background

In 2020, 40.3 million Americans were diagnosed with one or more substance use disorders (SUDs) (Substance Abuse and Mental Health Services Administration, 2021). Deaths involving multiple substances have steadily increased since 2014 and there was a 44% increase in opioid-related deaths from 2019 to 2020 alone [1, 2]. These concerning trends are accompanied by increased wounds and skin and soft tissue infections (SSTIs), which are cited as the most common reasons people who inject drugs (PWID) visit the Emergency Department (ED) and are considered risk factors for readmission and death [3–6]. The United States has seen a drastic increase in substance-related ED visits and acute hospital admissions in the past decade as a lack of access to primary care and specialty SUD treatment has positioned acute care settings as the primary access point to healthcare for many people who use drugs (PWUD) [7]. This increased healthcare contact creates opportunities to engage with people who use drugs, but these interactions will only be successful if clinicians are appropriately trained to address substance use.

Harm reduction is increasingly recognized as an essential part of healthcare for PWUD as it provides a set of evidence-based, practical strategies that aim to reduce the negative consequences associated with substance use [8–11]. Harm reduction as a larger concept and movement has its roots in many intersecting, community-driven social justice movements active in the late twentieth century in the United States [10]. Given this intentionally de-centralized and grassroots nature of harm reduction, there is no universally accepted definition for harm reduction theory, which is seen as both a strength and a weakness by advocates and critics alike [12, 13].

The six values of humanism, pragmatism, individualism, autonomy, incrementalism, and accountability without termination undergird the development of harm reduction principles and assist healthcare professionals in meeting the complex healthcare needs of PWUD [14]. These values are built on a belief in, and respect for, the rights of PWUD and are operationalized by the National Harm Reduction Coalition (NHRC) into eight guiding principles. These principles foremost 1) accept the reality of drug use and work to minimize its harmful effects rather than condemning them, and 2) understand drug use as a complex continuum of behaviors that range from severe use to total abstinence, acknowledging that some ways of using drugs are clearly safer than others (see Table 1) [10].

**Table 1:** Harm Reduction Principles from the National Harm Reduction Coalition

1. Accepts the reality of drug use and works to minimize its harmful effects rather than condemning them	
2. Understands drug use as a complex continuum of behaviors that range from severe use to total abstinence and acknowledges that some methods of using drugs are safer than others	
3. Positions the holistic wellbeing of individuals and communities as the primary measure of successful interventions and policies, not necessarily abstinence from substances	
4. Promotes the non-judgmental, non-coercive provision of services and resources to folks who use drugs to assist them in reducing attendant harm	
5. Ensures that people with lived experience of drug use have influential roles in the creation of programs and policies that serve and affect them	
6. Situates people who use drugs as autonomous agents of risk reduction and empowers communities with the information and resources they need to mutually support one another in ways that are tailored to their conditions of use	
7. Understands that social inequities such as poverty, class, racism, sexism, social isolation, and past trauma can impact people’s vulnerability to and ability to effectively respond to drug-related harm	
8. Does not disregard nor minimize the very real and tragic harms that can be associated with substance use	

The practical evidence-based clinical strategies derived from these principles include increasing naloxone availability, developing syringe service programs (SSPs), and implementing techniques that reduce the risks associated with drug use, such as overdose and transmission of HIV and HCV infection [14–17].

In 2019, ACGME guidelines began requiring graduate medical education (GME) programs to educate their residents on opioid use and an increasing number of undergraduate medical education (UME) curricula included topics on the prevention, diagnosis, and pharmacologic treatment of opioid use disorder (OUD) [18–21]. As of June 2023, the Substance Abuse and Mental Health Services Administration (SAMHSA) and Drug Enforcement Administration (DEA) have abolished the X-waiver that previously required physicians to declare intent to prescribe medication for opioid use disorder (MOUD). Now, the DEA requires all DEA-registered practitioners to complete a similar one-time, eight-hour training on the treatment and management of patients with OUD and SUDs, enforcing a standardized educational requirement for all medical trainees and further emphasizing the need for a well-educated workforce [22]. Medication treatments for SUDs reduce the risks associated with substance use and can be provided to patients as part of evidence-based, harm reduction informed services [23–26]. However, provider-level barriers remain a significant challenge in the provision of SUD services as there is widespread uncertainty among physicians about how to implement harm reduction strategies in clinical practice [27–31]. The most common issues reported are 1) a lack of clinician confidence or comfort in providing guidance and education to PWUD and 2) a lack of medical education and training needed for clinicians to develop these skills [32–35].

A survey of family medicine residents across three different institutions reported a gap between residents’ sense of responsibility for and their confidence in their abilities to conduct screening, brief intervention, and referral to treatment (SBIRT) [36]. For example, while 86.1% of surveyed residents reported feeling responsible for referring patients to buprenorphine or methadone clinics and SSPs, only 21.5% were confident in their ability to do so. Similarly, a study of internal medicine residents at an academic medical center in New York City reported 87% of respondents felt responsible for educating patients on overdose prevention and prescribing naloxone, but only 17% reported doing so in practice [37]. Development of recommendations to bridge the gap between trainee knowledge, confidence, comfort, and practiced behaviors first requires an evaluation of current curricula in both UME and GME to identify what educational approaches (i.e., curricular content, format, and evaluation) are already being used.

Past reviews examining medical education curricula on SUDs have either taken a global approach or solely focused on the use of MOUD [32, 38]. The present study conducts a systematic scoping review of peer-reviewed published curricula on harm reduction for substance use implemented by UME and GME programs in the United States and Canada. Our primary aims are to: 1) provide a comprehensive and detailed review of the content, format, and evaluations of these curricula, 2) develop a framework to describe a comprehensive approach to harm reduction medical education, and 3) propose additional harm reduction content topics for future consideration. By compiling a list of these publicly available harm reduction curricula published by UME and GME programs, we hope to facilitate the expansion of collaborative harm reduction education for physician-trainees and other health professional students and staff.

## Methods

Our systematic scoping review follows the Preferred Reporting Items for Systematic Reviews and Meta-Analyses extension for Scoping Reviews (PRISMA-ScR) [39].

### Protocol and registration

This review did not have a pre-registered protocol as PROSPERO does not register scoping review protocols.

### Eligibility criteria

To be included in this systematic scoping review, articles needed to fulfill the six eligibility criteria decided upon by our review team (see Limitations section to examine how these decisions affected our findings.) First, curricula must focus on harm reduction theory, harm reduction principles, or harm reduction practices related to substance use. As outlined previously, harm reduction theory does not have one universally accepted definition, so we relied on the principles proposed by the NHRC in Table 1 to guide our analysis. We operationalized these principles and our experience-based expertise to separate harm reduction curricula from purely substance-related curricula. For example, a curriculum that teaches residents how to administer naloxone in the ED would be considered substance-related but not harm reduction; as this skill is being taught as a job duty, not as a technique to nonjudgmentally meet patients where they are at. However, teaching residents how to distribute take-home naloxone to patients who arrive in the ED because of an overdose or who report substance use would be considered harm reduction.

Second, articles must describe curricula taught in UME or GME programs. Examples of curricula that would be excluded include continuing medical education (CME) and trainings hosted by community organizations. UME and GME are required for all licensed, board-certified physicians whereas specific CME courses and community-based training must be intentionally sought-out by interested parties. Our goal was to characterize the medical education curricula experienced by the largest number of trainees.

Third, curricula must be taught at programs within the United States or Canada. Despite the differences in healthcare policy and delivery, the uniformity of medical education standards upheld by the Liaison Committee for Medical Education (LCME) [40] and the ease of cross-border physician education and employment [41–43] urge us to consider both American and Canadian medical education programs when examining the educational experiences of physicians practicing in the United States. Curricula taught in programs outside of these two countries were excluded given their limited impact on practice within the United States and generalizability due to differences in UME or GME program structure.

Fourth, articles must be published in peer-reviewed journals. This excludes curricula that were never published or published in grey literature (e.g., conference abstracts, institution websites, press-release updates on curricula initiatives). Much of medical education curricula development is rooted in evidence-based, peer-reviewed research and one of our primary aims is to describe the current state of the high-caliber harm reduction resources medical educators have at their disposal. Additionally, many of our extraction variables discussed below (e.g., curricula format, educational content, outcomes tracked) are typically missing from these sources which would limit our ability to provide future educators with examples of detailed precedent.

Fifth, articles must be published in or after the year 1993. Although harm reduction principles and practices were utilized by a variety of grassroots movements throughout the mid-twentieth century, 1993 is considered the start of the modern-day harm reduction movement as this year marked the creation of a small working group which is now known as the NHRC [44].

Sixth, articles must be published in English. None of our authors can fluently read or write in languages other than English, so all articles written in languages other than English were excluded from this non-funded study.

### Information sources

Three databases were searched: PubMed, Scopus, and ERIC: Education Resources Information Center (Ovid). The journal MedEdPORTAL was also searched via its own site. Searches retrieved all records from the start of the database through Nov 22, 2021.

### Search

Search strategies were developed and tailored for each database or platform by Graduate Medical Education Librarian author ALA and author KRS. Keyword lists for medical education were developed using common colloquial terms for UME and GME (e.g., medical school, residency, medical curricula) and relevant subject headings from each of the databases. Initial keyword lists on the topic of harm reduction were developed using authors’ expertise and relevant subject headings from each of the databases. These initial lists were reviewed and finalized in consultation with an expert in harm reduction research who has a Doctorate in Public Health. Examples of harm reduction keywords used include ‘overdose prevention’, ‘syringe services programs’, and ‘injection site rotation’. Despite the increasing recognition of harm reduction in medical education, we expected that some UME and GME programs may discuss harm reduction practices but not label them as such. To account for this, we included terms that targeted broader concepts such as substance use and the Medical Subject Heading (MeSH) for Substance-Related Disorders. Full search strategies and keyword lists for both harm reduction and medical education are available as an additional file (see Additional file 1). Databases without an educational focus (PubMed and Scopus) were searched using the keyword lists for both medical education and harm reduction, while databases or platforms with an educational focus (ERIC and MedEdPORTAL) were searched using only the keyword lists for harm reduction. Records from all four sources were imported into the EndNote 20 reference management tool (Clarivate, Philadelphia, PA, USA) for deduplication. Duplicates identified based on matching DOI or matching title, author, year, and journal were excluded.

### Selection of sources of evidence

Two reviewers (KRS and NKS) independently screened records based on title and abstract using the eligibility criteria outlined above in a standardized spreadsheet. Screening decisions were reviewed in regular group meetings and disagreements were reconciled by both reviewers. After title/abstract screening, manuscripts of all articles selected for full-text screening were obtained. Full-text screening was performed independently by authors KRS and NKS and reviewed in regular meetings. Disagreements were discussed between the two reviewers with additional input from a third author (ELK) when necessary.

### Data charting process and data items

Microsoft 365 Excel was used for data extraction and storage. Authors KRS, NKS, and ELK created a preliminary standardized list of variables for data extraction including reason for curriculum creation, institution, trainee population, format, curricular components, educational content, outcomes tracked, and public availability of the curriculum. These extraction variables were selected because they align with the primary aims of this review to provide detailed evidence on the harm reduction educational content taught in these curricula and the format in which those curricula were taught and evaluated. However, the content on harm reduction principles were reported with limited context so this variable had to be distilled to a binary yes/no instead of a descriptive inclusion of each of the eight principles offered by the NHRC. Authors KRS and NKS tested this template on one agreed-upon article from the final sample and, after independent data extraction, reconvened to revise the list of variables and agree upon a uniform notation style. The remaining articles were then split evenly between authors KRS and NKS for independent data extraction. If relevant data arose throughout independent review that did not fit into one of the agreed upon variables, reviewers would add a new variable to the extraction list. At the end of independent data extraction, the list of variables was merged between reviewers and updated to encompass all available data in the articles. Each author then independently reviewed their originally assigned articles a second time to ensure that no data were missing for the finalized list of variables that are reported in the Results. In a final data meeting, authors KRS and NKS reviewed all data extracted from all articles together to ensure that documentation notation was uniform and fully understood by both parties.

### Synthesis of results and framework development

Authors KRS, NKS, and LCW conducted data synthesis using 1) descriptive statistics for all reported variables and 2) additional qualitative analysis of the variable ‘educational content’ using summative content analysis. Summative content analysis is a qualitative research technique used to identify, quantify, and contextualize discrete content topics [45]. As described previously, authors KRS and NKS identified the educational topics covered in each article and documented how many times each topic arose in all curricula. Qualitative summative content analysis is different from quantitative descriptive statistics in that it goes beyond counting appearances of a topic and uses latent content analysis to interpret the context in which these topics are used. Authors KRS and NKS initially grouped some items into small clusters based on superficial similarities (e.g., skills, physical resources, abstract principles), but this method proved insufficient when too many topics were left alone. In reviewing this initial effort, the authors determined that lenses of both medical education and harm reduction had to be used at the same time to categorize topics. Guided by author LCW, a medical educator, harm reduction expert, and board-certified family medicine and addiction medicine physician, authors then conducted an exercise to categorize topics based on how we would present them to a class of medical students who had no prior knowledge of or experience with substance use and harm reduction. As larger categories started to emerge, authors identified the common characteristics shared by that group of topics and began to develop themes based on them; these themes were then reviewed by author ELK and placed on an educational spectrum. Finally, authors consulted with colleagues in both harm reduction and clinical medication education to gain expert consensus on the final product of this synthesis.

## Results

### Sample

**Fig. 1:**
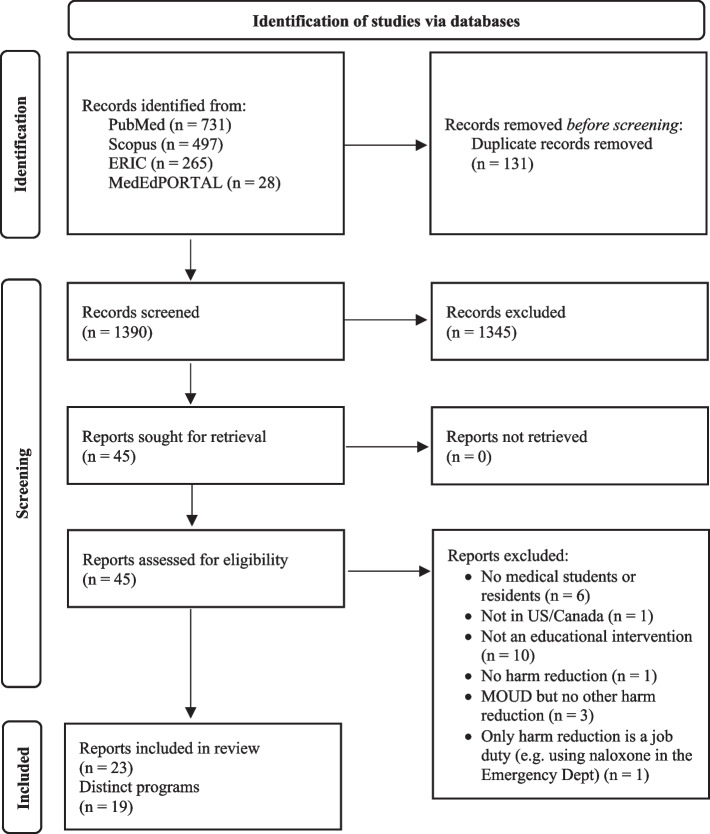
PRISMA flow diagram of the literature search, screening, and selection process conducted in November 2021

1521 records were pulled from PubMed, Scopus, ERIC, and MedEdPORTAL. Records were imported into EndNote and deduplicated, leaving 1390 unique records which were exported to an Excel spreadsheet for screening. After title/abstract screening for UME or GME programs and harm reduction principles and practices, 45 records were retrieved for full text screening. During full text screening, 22 articles were excluded based on various criteria outlined in Fig. 1, leaving a final sample of 23 articles reporting on 19 distinct curricular programs that are all located within the United States.

States included CA, CT, FL, MA (4), MD, MI, MO, NC, NY (3), OH, PA (2), RI, and VA. See Table 2 for summary descriptions of the 19 distinct curricula described by these articles [46–68].

**Table 2:** Content, format, and evaluation characteristics of 19 distinct harm reduction curricula

Study	Institution	Trainees	Format	Curricular Components	HR Principles ^a^	Content	Outcomes	Curriculum Availability
Berland 2017 and 2019 [46, 47]	New York University Grossman School of Medicine	MS1	-Mandatory -30 min. -In-person synchronous & online asynchronous	Didactics, small group discussion, teach-back, naloxone demonstration, self-evaluation	No	Naloxone, Good Samaritan laws, overdose etiology/physiology, overdose risk, overdose identification, identification of at-risk patients	1) Modified OOKS (*pre/post*)* 2) Modified OOAS (*pre/post*)* 3) Modified MCRS (*pre/post*) 4) Training feedback	Partially Available in article
Donohue 2021 [48]	University of Maryland Medical Center	FM and pediatrics PGY1	-Mandatory -Time NR -In-person synchronous	Didactics, small group discussion, SPs, simulated scenarios, case studies, naloxone demonstration	No	Naloxone, nonjudgmental language and stigma, overdose identification, patient screening, person-centered goals and MI, MOUD, withdrawal identification and treatment, infectious complications	1) Program-created scales on comfort treating patients with OUD (*pre/post*)*	Not Available
Funke 2021 [49]	Duke University Hospital Emergency Department	EM PGY1-PGY3, PAs, nurses, attending physicians	-Requirement NR -4h hr. -In-person synchronous & online asynchronous	Didactics, small group discussion, patient panel, local organizations, EHR training, self-evaluation	No	Naloxone, related laws, overdose etiology/physiology, overdose identification, SBIRT and identification of at-risk patients, MOUD, withdrawal identification and treatment, myth-busting	1) Program-created survey on attitudes towards patients with OUD (*pre/post-3 months*)* 2) Reasons given to not prescribe naloxone (*post-2 months*) 3) Percent of at-risk patients prescribed naloxone (*pre/post.post-1 year*)*	Partially Available in article supplementary materials
Goss 2021 [50]	Drexel University College of Medicine	MS1	-Voluntary -2 hr. -In-person synchronous & online asynchronous	Didactics, small group discussion, videos, naloxone demonstration, patient panel	Yes	Overdose prevention, naloxone, safer injection, SSPs, fentanyl test strips, nonjudgmental language and stigma, Good Samaritan laws, overdose etiology/physiology, overdose risk, overdose identification, MOUD	1) Modified OOAS (*pre/post*)* 2) Modified OOKS (*pre/post*)*	Not Available
Han 2017 [51]	University of Pittsburgh Medical Center: St Margaret Family Medicine Residency	FM PGY1-PGY3, fellows, pharmacy residents, social workers, faculty	-Mandatory -Time NR -In-person synchronous	Didactics, naloxone demonstration, EHR training	No	Naloxone, overdose risk, overdose identification, opioid prescription guidelines, MI	1) Program-created scales on learner attitudes and comfort (*pre/post-6 months*)* 2) Number of naloxone kits prescribed 3) Number of opioid overdoses reversed 4) Patient willingness to obtain and use naloxone from providers (*post-2 months*)	Not Available
Hargraves 2019 [52]	University of Cincinnati: Family and Community Medicine Residency	FM PGY1 and PA students	-Mandatory -2 hr. -In-person synchronous	Didactics, videos, naloxone demonstration	No	Naloxone, Good Samaritan laws, overdose etiology/physiology, overdose identification, identification of at-risk patients	1) Program created scales on learner knowledge, attitudes, and self-efficacy (*pre/post*)* 2) Learner satisfaction with various curricular components (*post*)	Fully Available in article
Jack 2018 [53]	Harvard Medical School	MS3 (pilot) MS1 (long-term program)	-Mandatory (long-term program) -5 min. -In-person synchronous	Naloxone demonstration	No	Naloxone, overdose etiology/physiology, overdose risk, overdose identification	No evaluation of student learning was reported	Not Available, but based on AHA Opioid Associated Life-Threatening Emergency Algorithm
Jawa 2020 [54]	Boston Medical Center at Boston University School of Medicine	IM PGY1 (pilot) Medical and surgical PGY and fellows (expanded program)	-Voluntary (expanded program) -15 min. -In-person synchronous	Didactics, small group discussion, naloxone demonstration	No	Naloxone, Good Samaritan laws, overdose etiology/physiology, overdose identification	1) Comfort administering naloxone (*pre/post – 1 month*)* 2) Comfort counseling patients on naloxone use (*pre/post – 1 month*)* 3) Naloxone prescribing behavior (*pre/post – 1 month*)	Fully Available in MedEd Portal
Jawa 2021 [55]	Boston University School of Medicine	MS1, MS2, medical masters students, PA students, dental students	-Voluntary -2 hr. -In-person synchronous	Didactics, naloxone demonstration, local organizations	Yes	Overdose prevention, naloxone, safer injection, SSPs, overdose etiology/physiology, overdose risk, overdose identification, MOUD, infectious complications	1) Comfort with harm reduction ideology (*pre/post*)* 2) Knowledge on safe injection technique (*pre/post*)* 3) Comfort and knowledge on administering naloxone (*pre/post*)*	Not Available
Klapheke 2017 [56]	University of Central Florida College of Medicine	MS3 psychiatry clerkship	-Mandatory -30-50 min. -In-person synchronous or online asynchronous	Didactics, small group discussion, videos, case studies, naloxone demonstration, self-evaluation	No	Overdose prevention, naloxone, overdose etiology/physiology, overdose risk, overdose identification, opioid prescription guidelines, patient screening, person-centered goals, MOUD, withdrawal identification and treatment	1) Program-created survey on confidence in knowledge of risk reduction, patient education (*post*) 2) Evaluation of module as educational tool (*post*)	Fully Available in MedEd Portal
Monteiro 2017 and 2017 [57, 58]	Warren Alpert Medical School at Brown University	MS2, nursing students, physical therapy students, social work students, pharmacy students	-Requirement NR -4 hr. -In-person synchronous	Small group discussion, SPs, videos, case study, naloxone demonstration, patient panel	No	Naloxone, non-judgemental language, overdose risk, overdose identification, SBIRT, MOUD	1) Learner self-reflection (*post*) 2) OOKS (*pre/post-12 weeks*)* 3) Satisfaction survey (*post*) 4) Curricular component Likert scale evaluations (*post*)	Fully Available in MedEd Portal
Moses 2021, 2022, and 2022 [59–61]	Wayne State University School of Medicine	MS1, MS3	-Mandatory -1 hr. -In-person synchronous	Didactics, naloxone demonstration	Yes	Overdose prevention, naloxone, Good Samaritan laws, overdose etiology/physiology, overdose risk, overdose identification, myth busting	1) OOKS (*pre/post*)* 2) OOAS (*pre/post*)* 3) MCRS for SUDs (*pre/post*) 4) NaRRC-B (*pre/post*)* 5) Learner satisfaction and experience (*post*)	Not Available
Oldfield 2020 [62]	Yale School of Medicine	MS3 primary care and psychiatry clerkship	-Mandatory -75 min. -In-person synchronous & online synchronous	Didactics, train-the-trainer, videos, naloxone demonstration	Yes	Overdose prevention, naloxone, safer injection, SSPs, non-judgmental language, overdose etiology/physiology, overdose risk, overdose identification, identification of at-risk patients	1) OOKS (*pre/post-6 wks*)* 2) OOAS (*pre/post-6 wks*)* 3) MCRS (*pre/post-6 wks*)* 4) Self-reported clinical behavior (*pre/post-6 wks*) 5) Anonymous satisfaction survey (*post*)	Fully Available in article appendix
Parish 2013 [63]	Albert Einstein College of Medicine & Montefiore Medical Center	IM and FM PGY3	-Mandatory -2.5 hr. -In-person synchronous	OSCEs, SPs, small group discussion, self-evaluation	Yes	Overdose prevention, safer infection, SSPs, non-judgemental language and stigma, overdose risk, SBIRT and identification of at-risk patients, person-centered goals, MI, MOUD, withdrawal identification and treatment	1) Faculty and SP evaluation of resident communication, SUD assessment and management 2) Evaluation of OSCE as educational tool	Fully Available in article
Riazi 2021 [64]	REACH Program at Mt Sinai Hospital & Icahn School of Medicine at Mt Sinai	MS1, IM PGY, nursing, health staff	-Mandatory -30-60 min. -In-person synchronous	Didactics, train-the-trainer, naloxone demonstration, local organizations	No	Overdose prevention, naloxone, SSPs, person-centered language, Good Samaritan laws, overdose etiology/physiology,overdose risk, overdose identification	No evaluation of student learning was reported	Fully Available in article supplemental materials
Taylor 2018 [65]	Beth Israel Deaconess Medical Center at Harvard Medical School	IM PGY	-Mandatory -2 hr. -In-person synchronous	Didactics, small group discussion, videos, naloxone demonstration, EHR training	No	Naloxone, safer injection, identification of at-risk patients	1) Inpatient & outpatient naloxone prescriptions (*pre/post-3 months*)* 2) Program-created survey on learner knowledge (*pre/post*) 3) Program-created survey on learner attitudes (*pre/post*)*	Partially Available in article
Tringale 2017 [66]	Keck School of Medicine of USC & Homeless Healthcare Los Angeles	MS1	-Voluntary -4 hr. -In-person synchronous	Didactics, small group discussion, shadowing, patient interaction, local organizations, self-reflection	Yes	Safer injection, SSPs, nonjudgmental language and stigma, person-centered goals, MOUD	1) Learner evaluation of experience and learning objectives (*post*) 2) Open-ended reflection (*post*)	Not Available
Winograd 2017 [67]	Veterans Affairs St. Louis Healthcare System	MS4 IM rotation, PGY (mostly primary care), attending psychiatrists, NPs, PAs, clinical pharmacists	-Voluntary -25-40 min. -In-person synchronous	Didactics, naloxone demonstration	No	Overdose prevention, naloxone, SSPs, overdose etiology/physiology, overdose risk, overdose identification, identification of at-risk patients, MI	1) Modified OOKS (*pre*) 2) Modified OOAS (*pre*) 3) Monthly naloxone prescriptions (*pre/post – 10 months*) 4) # naloxone prescribers (*pre/post – 10 months*)	Fully Available through corresponding author
Zanjani 2020 [68]	Virginia Commonwealth University & Richmond Health and Wellness Program	MS, nursing, pharmacists, psychologists, social workers, allied health professionals, faculty	-Mandatory -1 hr. -In-person synchronous & online asynchronous	Didactics, small group discussion, SPs, case studies,	No	Overdose prevention, overdose risk, opioid prescription guidelines, SBIRT, MI	Program-created scales on (*pre/post*): 1) Learner knowledge* 2) Learner perceived skills* 3) Learner motivations	Not Available

^a^The Harm Reduction Principles column in this table denotes which of the 19 distinct curricula either 1) explicitly report teaching harm reduction as ‘principles’, ‘theory’, ‘framework’, ‘ideology’, or ‘model’ or 2) despite not naming it as such, teach harm reduction as a critical thinking framework and not just as a set of static practices. These designations were assigned by our authors based on their expertise in harm reduction and medical education

*Denotes significant results

### Reasons for curriculum development

Of the 19 distinct programs, 16 (84%) cited the worsening opioid overdose epidemic as the primary impetus for their curricula creation. The second most frequently cited reason was the lack of standardized medical education curricula on harm reduction for OUD and other SUDs (*n* = 14, 74%). About half of the programs also noted that this educational gap along with physicians’ discomfort with harm reduction practices have left physicians unprepared to support patients who actively use substances (*n* = 9, 47%). Programs also discussed the increased vulnerability of their patient populations, the growing evidence-basis for MOUD and harm reduction practices, and a lack of consistency in the validity and implementation of existing tools meant to screen for and assess harm reduction behaviors as motivation for creating novel educational curricula. Finally, three programs explicitly noted that students vocally advocated for more curricular time devoted to these topics (*n* = 3, 16%).

### Trainees

Ten (53%) of the 19 distinct curricula trained only undergraduate medical students, 7 (37%) trained only graduate medical trainees, and 2 (10%) trained both. Of these programs, 8 included students from other health professions including nursing (*n* = 5, 26%), pharmacy (*n* = 4, 21%), physician assistants (*n* = 4, 21%), social work (*n* = 3, 16%), physical therapy (*n* = 1, 5%), and dental (*n* = 1, 5%). Additionally, 4 (21%) programs included faculty or attending physicians in their trainee population. The number of physician trainees and additional healthcare students and professionals (e.g., nurses, physical therapists, physician assistants, pharmacists) participating in each distinct program ranged from 26 to 540 with an average of 199.5 participants. Most programs (*n* = 10, 53%) trained under 200 participants. Three programs (16%) did not report their number of participants. The interventions that targeted learners during a clerkship rotation or residency were most commonly taught in family medicine (*n* = 6), internal medicine (*n* = 5), and psychiatry (*n* = 4).

### Curricula format and components

Seventeen of the 19 distinct educational institutions (89%) reported creating their own curricula based on or in collaboration with Substance Abuse and Mental Health Services Administration (SAMHSA), Center for Disease Control and Prevention (CDC), Health Resources and Services Administration (HRSA), San Francisco Dept. of Public Health (SFDOPH), and the National Harm Reduction Coalition (NHRC). Two programs (11%) used materials previously created by the American Heart Association (AHA) and the NYC Department of Health and Mental Hygiene’s *Save a Life- Carry Naloxone* training*.* Interventions were facilitated by faculty members with appointments in a variety of specialties, health professionals with expertise in SUDs, local harm reduction agencies, patients with lived experience and, in some cases, by medical trainees themselves. The experience level of these facilitators was mostly unspecified. Attendance requirements among programs varied as 12 institutions (63%) reported that their curricula were mandatory, 5 (26%) were voluntary, and 2 (11%) were unspecified. All programs provided in-person synchronous instruction with one-third also incorporating online curricular components like modules or videoconference classes. Length of instruction ranged from 5 minutes to 4 hours, but most programs lasted between 30 minutes and 2 hours (*n* = 12, 63%). The most common curricular components included didactics (*n* = 16, 84%), naloxone demonstrations (*n* = 15, 79%), and small group discussions (*n* = 11, 58%). Many programs also used a combination of videos (*n* = 6, 32%), standardized simulated clinical encounters/scenarios (e.g., Objective Structured Clinical Encounters, Standardized Patients, mannequins) (*n* = 4, 21%), case studies (*n* = 4, 21%), and patient panels/interviews (*n* = 4, 21%), although these were reported less frequently.

### Public availability

Overall, 8 curricula (42%) are fully publicly available through the journal of publication or by request of the authors, 3 (16%) are partially available, and 8 (42%) are neither online nor explicitly available upon request.

### Evaluation

Seventeen of the 19 distinct programs (89%) conducted evaluations on the effectiveness of their curricula using a variety of techniques. Most programs focused their evaluations on changes in self-reported knowledge and attitudes. Fifteen (79%) used program-created surveys that were administered to learners to assess aspects of learning such as comfort or confidence applying learned concepts to patient care (*n* = 6, 32%), knowledge of (*n* = 4, 21%) and attitudes towards (*n* = 4, 21%) treating patients with OUD, and self-reported naloxone prescribing behaviors (*n* = 2, 11%). Six programs incorporated a modified version of previously validated surveys, including the Opioid Overdose Knowledge Scale (*n* = 6, 32%), Opioid Overdose Attitudes Scale (*n* = 5, 26%), Medical Condition Regard Scale (*n* = 3, 16%), and the Naloxone-Related Risk Compensation Beliefs Scale (*n* = 1, 5%). Finally, 4 programs collected objective data measuring the impact of their interventions, including the number of naloxone prescriptions or prescribers (*n* = 4, 21%) and number of opioid overdose reversals reported by patients (*n* = 1, 5%). Separately, ungraded learner feedback on general opinions of the curricula were obtained in 8 programs (42%).

Most programs used pre/post-intervention assessments and reported statistically significant positive changes in knowledge, attitudes, confidence, and self-efficacy as they relate to working with PWUD (*n* = 13, 68%). However, of the 16 programs that conducted a post-intervention evaluation, only seven (37%) included a follow-up evaluation of more than 1 month after the intervention.

### Educational content description and summative content analysis

**Fig. 2:**
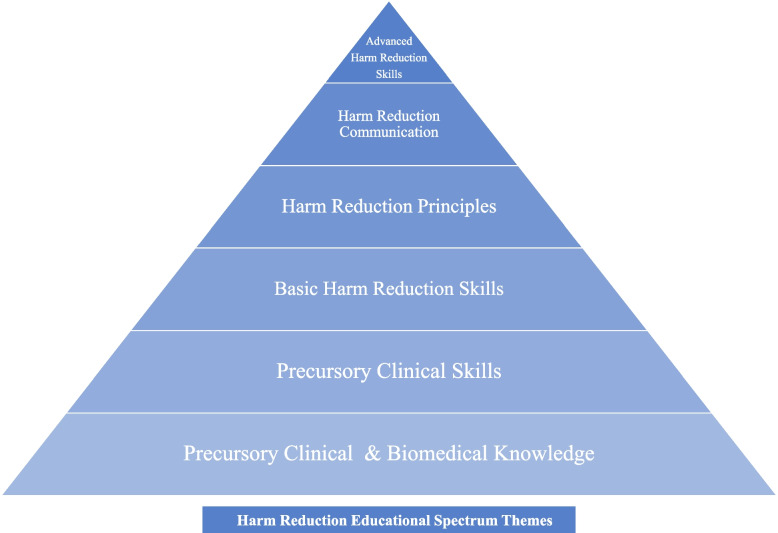
The Harm Reduction Educational Spectrum (HRES) Framework. (The HRES Framework developed by the authors describes how the discrete content topics found during a scoping review of 19 distinct harm reduction curricula taught in undergraduate and graduate medical education programs in the United States + Canada build upon one another and ultimately require an understanding of Harm Reduction Principles for full progression. Completed in Nov 2021.)

**Fig. 3:**
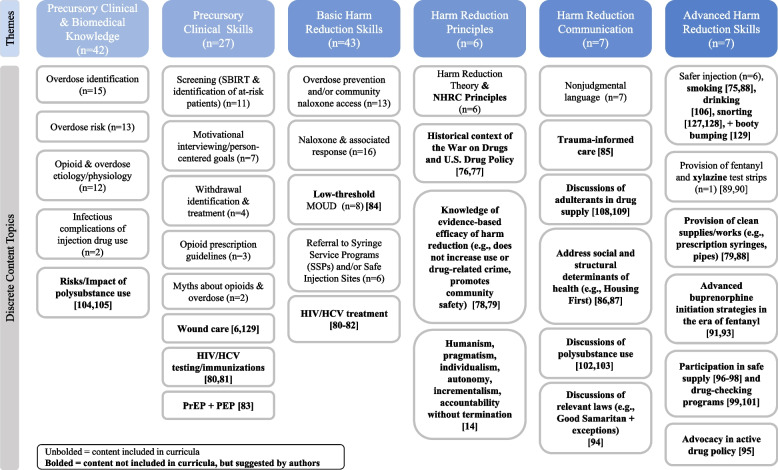
Results of educational content analysis and application of Harm Reduction Education Spectrum (HRES) framework. (The discrete content topics included in the educational content of 19 distinct harm reduction curricula and how many times each of those topics arose across programs are presented un-bolded. These topics have been organized into the HRES framework which was developed by the authors with the themes identified during analysis to summarize the topics found during the scoping review. Content topics that were not explicitly reported by any programs but are additional topics that our authors believe should be included in a robust harm reduction education are presented in bold.)

We identified 17 discrete content topics that mapped onto six larger themes: *Precursory Clinical & Biomedical Knowledge*, *Precursory Clinical Skills*, *Basic Harm Reduction Skills, Harm Reduction Principles*, *Harm Reduction Communication*, and *Advanced Harm Reduction Skills*. We classified the different educational themes along a continuum of knowledge from basic understanding of substance use to clinical skills requiring mastery of foundational concepts and a dedication to harm reduction principles; we propose this as the Harm Reduction Educational Spectrum (HRES) framework (see Figs. 2 and 3) [69].

The themes of *Precursory Clinical & Biomedical Knowledge* and *Precursory Clinical Skills* include opioid/substance-related content that could be taught in any curricula focusing on substance use. The content topics identified as *Precursory Clinical & Biomedical Knowledge* include overdose identification (*n* = 15, 79%), overdose risk (*n* = 13, 68%), overdose etiology/physiology (*n* = 12, 63%), and infectious complications of injection drug use (*n* = 2, 11%). Overall, these topics were referenced a total of 42 times across 19 distinct curricula. Referenced a total of 27 times, *Precursory Clinical Skills* include screening (SBIRT & identification of at-risk patients) (*n* = 11, 58%), motivational interviewing/person-centered goals (*n* = 7, 37%), withdrawal identification & treatment (*n* = 4, 21%), opioid prescription guidelines (*n* = 3, 16%), and myths about opioids & overdose (*n* = 2, 11%). Although many of these skills could be practiced through a harm reduction lens, they were reported with limited context which led the authors of this review to categorize them conservatively as though they were not.

The theme of *Basic Harm Reduction Skills* includes skills and resources that implicitly recognize the possibility of ongoing substance use in patients and decrease the physical risks associated with substance use. However, these are conceptually distinct from more advanced skills because they do not require a practical understanding of harm reduction principles which offer insight into how people obtain, prepare, and use drugs. From the 19 distinct curricula, authors identified three content topics that fit within this category: naloxone & associated response (*n* = 16, 84%), overdose prevention & community naloxone access (*n* = 13, 68%), MOUD (*n* = 8, 42%), and referral to SSPs and/or Safe Injection Sites (*n* = 6, 32%). Together, these items were referenced a total of 43 times.

The theme of *Harm Reduction Principles* includes discrete content topics that help teach harm reduction as a critical thinking lens that can be broadly applied to a variety of relevant clinical situations and not just a set of static practices. Programs that taught harm reduction as a critical thinking framework or explicitly reference it as a ‘theory’, ‘framework’, ‘ideology’, or ‘model’ were included (*n* = 6, 32%). This theme is a foundational piece of harm reduction education as it is what separates a static skillset from one that can adapt to new challenges and an ever-changing drug landscape. Harm reduction principles provide the foundation for the critical application of many advanced harm reduction skills; while there is no requirement to progress linearly through the HRES framework, we believe that trainees without an understanding of the content topics in this theme are likely to have a narrower, less flexible skillset than their colleagues who do.

*Harm Reduction Communication* includes examples of how physicians can apply harm reduction framework to interpersonal skills and communication with patients. The topic of nonjudgmental language fell into this category and was taught in seven of the 19 distinct curricula (*n* = 7, 37%).

The theme *Advanced Harm Reduction Skills* follows the same fundamental definition as *Basic Harm Reduction Skills* but differs in that the skills included here explicitly recognize the possibility of ongoing substance use in patients and include a practical understanding of harm reduction principles which offer insight into how people obtain, prepare, and use drugs. Additionally, these skills are underpinned by an advanced knowledge of the historical, structural, and contextual factors that shape public perceptions of PWUD and substance use policy, which in turn affect our patients’ self-perceptions and receptivity to healthcare. Of the content topics taught in reviewed curricula, safer injection (*n* = 6, 32%) and fentanyl test strips (*n* = 1, 5%) were categorized here and referenced a total of seven times.

After mapping the current curricula onto the HRES framework, we expanded our list of content topics to include items that would ideally also be part of a comprehensive curriculum (see Fig. 3). These recommendations were based on best-practices noted in the literature and the expertise developed by our authors while immersed in both clinical and non-clinical harm reduction spaces.

## Discussion

The overarching goals of this study were to identify the core components of existing UME and GME curriculum on harm reduction and use these to develop a framework to guide the approach of future curriculum development. With no AAMC or ACGME approved guidelines/materials on harm reduction, each of the 19 distinct programs reviewed here created and implemented a curriculum with little precedent to build on. As demonstrated by the uniformity of motivations behind curricula development, medical educators see an urgent need to address this deficit and develop and disseminate evidence-based harm reduction curricula. Echoing the literature review findings of Muzyk et al. and Kothari et al., we recommend that educators expand their focus to include harm reduction principles, communication, and skills and robust evaluation of trainees’ understanding and use of these techniques (see Table 3) [32, 70].

**Table 3:** Recommendations for future harm reduction curricula taught in undergraduate and graduate medical education programs

Recommendation	Corresponding Finding	Rationale
1. Include harm reduction principles in curricula	-Of the 19 distinct curricula reviewed, only six report explicitly teaching harm reduction as a set of principles, theory, or framework -85% of the content taught falls on the introductory side of the Harm Reduction Educational Spectrum (HRES) developed by the authors, before the point where skill progression benefits from an understanding of harm reduction framework	-Harm reduction principles offer the critical thinking skills foundational for understanding and applying more advanced harm reduction strategies -They emphasize the importance of other health principles necessary for working with PWUD [12] -They will help mitigate the lag in medical education that occurs when trying to characterize a fluctuating health crisis because instead of teaching students a set of static practices it encourages students to apply harm reduction as a framework
2. Include non-opioid (e.g., cocaine, psychostimulant, alcohol) related harm reduction	-None of the reviewed curricula report discussing the importance of harm reduction for substances other than opioids	-With rising rates of fentanyl adulteration in the unregulated drug supply [71, 72], our trainees need to be prepared to discuss opioid-related harm reduction (e.g., overdose identification, naloxone) with patients who use any type of drug -Morbidity and mortality related to stimulant use has increased since 2014 [2]
3. Include more advanced harm reduction skills	-Of all the discrete content topics taught across 19 distinct curricula, those that fell into the theme of Advanced Harm Reduction Skills (safer injection & fentanyl test strips) were only referenced 7 times; this amounts to 5% of curricula time	-Ensures that trainees are provided with a full picture of harm reduction practices -Adds tools to trainee’s toolbox; trainees cannot practice these skills or refer patients to other locations where they can receive these services without knowing they exist
4. Prioritize patient engagement through hands-on learning and center the experiences and expertise of PWUD in both content creation and instruction	-Only 4 programs report using patient panels or interviews as a component of their curricula format -No programs explicitly report consulting patients with substance use disorders or community members with lived experience using substances	-Harm reduction necessitates individualization; it is not a one-size fits all approach [10] -Demonstrates the variety of ways harm reduction can be operationalized in different spaces -Ensures the content taught is accurate and relevant -Further humanizes and destigmatizes the opioid epidemic
5. Prioritize the demonstration of critical application skills with existing public resources and provide trainees with opportunities to practice these skills in harm reduction-based standardized clinical encounters	-The only critical application skill reportedly taught was non-judgmental language which amounted to 5% of curricula time (*n* = 7) -Four of the 19 distinct curricula utilized standardized simulated clinical encounters/scenarios (e.g., Objective Structured Clinical Encounters/OSCEs, Standardized Patients/SPs, mannequins)	-There are not many positive, non-alienating examples of conversations about substance use given the stigma towards PWUD -Provides trainees with the words they need to have difficult conversations -Gives trainees hands-on practice that will help bridge the gap between knowledge and behavior
6. Conduct both process and outcome evaluations of curricula, utilize validated measures, collect qualitative and quantitative data, and conduct assessments pre-, post-, and long-term post-intervention	-Of the 17 programs that conducted evaluations of their curricula, 15 focused on comfort/confidence in applying concepts; only 6 of these use validated scales -Quantitative data was collected in only 4 programs (21%) -Only 7 (37%) programs conducted a post-intervention assessment more than 1-month after the intervention	-Validated measures support uniform comparison of curricula and their impact on learners -Qualitative and quantitative data can assess learner knowledge/attitudes/comfort/behavior and downstream patient outcomes -Process evaluations and longitudinal assessment allow for targeted interventions and iterative curricula improvement (e.g., identifying whether learners don’t understand/agree with a topic vs they don’t know how to implement what they have learned) [73, 74]
7. Publish programs' harm reduction curricula and utilize interdisciplinary expertise	-Only 8 programs (42%) are fully publicly available through the journal of publication or by request of the authors	-Many programs lack content experts in harm reduction, publishing your curricula encourages collaboration and reduces the barrier to entry for programs who want to begin implementing harm reduction education -Truly robust harm reduction curricula are interdisciplinary; public health, social work, and sociology/anthropology are all crucial

Although harm reduction as a set of practices has garnered both national [10, 75–77] and international [78, 79] interdisciplinary support, we suggest medical educators expand the scope of their curricula to ensure that harm reduction principles are taught as well. With 85% of the discrete educational content topics identified in this review falling into the first 3 themes (*Precursory Clinical & Biomedical Knowledge*, *Precursory Clinical Skills*, and *Basic Harm Reduction Skills*) of our iteratively developed Harm Reduction Educational Spectrum framework, we found that most programs emphasize introductory harm reduction skills without teaching harm reduction principles and history (see Fig. 3). This narrows learners’ skillsets because many advanced harm reduction practices explicitly recognize and require a basic understanding and curiosity about patients’ ongoing substance use and are best practiced with an understanding of the historical context and principles undergirding them. While certain topics have a natural progression to their development (e.g., providing MOUD requires an understanding of opioid & overdose etiology/physiology), we want to emphasize that content topics were categorized based on their relationship to harm reduction principles, not their relationship to each other. Our HRES framework is to be used within the context of UME and GME instruction, but there are various ways trainees may learn this material (e.g., personal experience, non-medical training programs) and the order in which they acquire skills may not always be linear. Our hope is that this framework can emphasize the interconnecting, and sometimes progressive, relationships between harm reduction knowledge/strategies without restricting educators and learners to a prescribed structure. The best harm reduction curriculum is one that meets the unique needs of learners and the communities they care for.

In reviewing the HRES framework, foundational content topics we encourage programs to dedicate curricular time to include harm reduction theory & NHRC principles, historical context on the War on Drugs and United States’ drug policy [80, 81], knowledge of the evidence-based efficacy of harm reduction (e.g., does not increase use or drug-related crime, promotes community safety, increases treatment engagement) [82, 83], and the Hawk et al. values that support integration of harm reduction into trainees’ bedside manner and care-plans [14]. These foundations will emphasize the importance of implementing known clinical strategies as tools of harm reduction such as providing HIV & HCV testing/immunizations/treatment [84–86], PrEP & PEP [87], and low-threshold MOUD [88], using a trauma-informed approach to care [89], and addressing the social and structural determinants of health that impact our patients on an individual level (e.g., Housing First models) [90, 91]. More advanced harm reduction skills are distinct from those in our *Basic Harm Reduction Skills* theme as they include a practical understanding of harm reduction principles which offer insight into how people obtain, prepare, and use drugs and recognize the affect structural and contextual factors have on individuals’ daily lives. The provision of clean supplies/works [83, 92], fentanyl & xylazine test strips [93, 94], and advanced buprenorphine initiation in the era of fentanyl [71, 95, 96] are all evidence-based strategies that can be used in one-on-one clinical encounters with patients and merit inclusion in future curricula. Finally, trainees can use their expertise as medical professionals and community leaders to advocate for harm reduction-based drug policy [72, 97], safe supply [98–100], and drug checking programs [101–103] .

The scaffolding provided by understanding and applying harm reduction principles can help mitigate the inevitable lag in relevance of curricular content to the fluctuating realities of the drug market in the United States. This lag is evident in the curricula we reviewed as we did not find explicit discussion of polysubstance use [73, 74, 104, 105], content unique to substances other than opioids (e.g., cocaine, methamphetamines, alcohol) [106, 107], or the increasing adulteration of the unregulated drug supply with fentanyl and xylazine, which are all current and rapidly evolving issues [108–110]. Collaborating directly with PWUD on curricula content creation and instruction (e.g., patient panels, patient-ran education sessions) can ensure the relevance and timeliness of curricula; strategies that were overall underutilized in the articles reviewed. Becoming trusted sources of information for reducing the harm associated with substance use requires that trainees nurture community partnerships and recognize the importance of patients’ anecdotal experiences, especially in situations where medical literature is lacking or lagging. Additional relevant subject matter in this area could include identifying/responding to stimulant overamping [107], testing non-opioid substances for fentanyl, and application of harm reduction framework to all types of substance consumption including alcohol (see Fig. 3) [111–114]. Unfortunately, the inclusion of harm reduction principles and practices in substance use curricula does not necessarily address the gap in trainee-reported knowledge/attitudes towards harm reduction and clinical behaviors [36, 37]. Of the 19 distinct curricula reviewed, didactics and small group discussion comprised most of the instruction with 4 programs employing standardized simulated clinical encounters/scenarios (e.g., OSCEs, SPs, mannequins). As with other topics that are similarly stigmatized or difficult to operationalize (e.g., serious illness, social determinants of health, LGBTQ health), we suggest that programs prioritize demonstrating these conversations and creating opportunities for trainees to practice them [115–117]. Programs could create a list of harm reduction-based clinical probes and demonstrate when/how to use them as well as utilizing publicly available resources, such as NIDA’s Words Matter project and Dr. Kimberly L. Sue’s clinical case study on The Curbsiders Addiction Medicine podcast [11, 118]. Inspired by the Sexual Orientation/Gender Identity Data Collection Demonstration videos created by the National LGBTQIA+ Health Education Center [119], our team at Thomas Jefferson University produced an Introduction to Harm Reduction in Healthcare video series in collaboration with PWUD [120]. These videos demonstrate how healthcare staff can discuss urine drug screen results and safer injection, approach phlebotomy with PWID, and support patients who might be in withdrawal in a waiting room through a harm reduction lens. Incorporating harm reduction into standardized patient (SP) encounters offers a novel opportunity for trainees to operationalize what they have learned in a didactic setting in a way that builds experiential confidence [121]. Similarly, we encourage programs to provide diverse options for hands-on learning alongside didactics. Examples could include volunteering at a syringe service program, joining a street medicine team, shadowing at an MOUD clinic, or completing a clinical rotation in addiction medicine or infectious disease. There are ways to integrate this education into pre-clinical and clinical years of UME and evidence demonstrates that community-based, longitudinal learning benefits both medical students and communities alike [122–124].

Finally, the evaluations conducted by curricular teams demonstrate significant increases in awareness and knowledge of trainees. However, they largely consist of program-created Likert scales [125], qualitative data, and assessment of short-term outcomes. Assessing quantitative, downstream process and outcome measures (e.g., learner behavior, EHR data on symptoms, patient satisfaction) at varying time points (pre-, post-, long-term post-intervention) will allow for standardization, comparison, and targeted improvement of curricula [126, 127].

We recognize that rigorous development, implementation, and evaluation of curricula requires significant curricular time, faculty, and resources. Educators can minimize the logistical burden of integrating harm reduction into established curricula by either spreading the content longitudinally across courses (e.g., Basic Life Support training, organ blocks, ‘doctoring’, pharmacology) or shifting the lens through which existing substance use curricula is already taught. In parallel, Windish et al. suggest that many programs may not have the resources to expand on their own substance use curricula given a lack of trained faculty content experts [128]. We encourage teams without content area expertise to expand and include interprofessional and interdisciplinary colleagues (e.g., psychologists, social workers, case workers, local SSP staff) and people with lived experience of substance use. Additionally, programs that do have harm reduction curricula should be supported by their institutions to evaluate and publish their curricula.

### Limitations

Many of our exclusion criteria impact the generalizability of our results as the present review is limited to English articles published in academic journals from January 1993 to November 22, 2021. The decision to exclude grey literature from our final sample hinged upon our goal to provide educators with evidence-based, peer-reviewed, and detailed content for future curricula development as previously discussed in our eligibility criteria. However, our narrowed scope inherently excludes curricula that have been developed but presented only at conferences, shared in more informal reports (e.g., institution websites, news articles, press-release updates on curricula initiatives), or never published. Similarly, our English-language inclusion criterion could have excluded additional articles written in French potentially contributing to the absence of Canadian program representation in our final sample and further limiting the generalizability of our findings for these programs.

Harm reduction is a rapidly changing field and the curricula described here may not reflect their current iterations due to publication timelines. The lack of fully readily available curricular materials for each distinct program also means that we relied on programs’ reports instead of evaluating curricula first-hand. Additionally, although the SAMHSA and DEA X-Waiver training was a standardized educational requirement for all physicians who intended to prescribe MOUD, this training did not meet our inclusion criteria and was subsequently excluded from our review. While the Consolidated Appropriations Act of 2023 has since abolished this requirement, the aforementioned training now required of all DEA-registered physicians should be included in future harm reduction and substance use curricula analyses [22, 129].

As outlined previously, our findings primarily focus on opioid-related harm reduction (e.g., overdose identification, naloxone) as no curricula reviewed explicitly mention harm reduction for other substances (e.g., cocaine, methamphetamines, alcohol). However, our inclusion criteria required that interventions include harm reduction related to drugs, meaning that curricula only focused on alcohol were excluded. Similarly, it is possible that programs discussed this content and did not explicitly report it.

Finally, the methods programs used to evaluate the efficacy of their instruction vary widely and this lack of standardization made comparisons of curricula quality and impact on learners and patient health outcomes difficult to assess. Therefore, we were unable to conduct a quality assessment of each article. We hope that our summary statistics provide some background for educators to structure future evaluations of their curricula. Identifying ways to ensure robustness and uniformity in evaluation for comparison and improvement is an opportunity for future exploration.

## Conclusions

Harm reduction principles and practices are increasingly recognized as crucial components of both undergraduate and graduate medical education. In our review of 19 distinct curricula, we found that most programs emphasize foundational substance use knowledge and introductory harm reduction skills without employing harm reduction as a framework. This narrows learners’ skillsets because many advanced harm reduction practices require a basic understanding of the reality of patients’ ongoing substance use and are best practiced with harm reduction principles. Timely topics such as polysubstance use [73, 74, 104, 105], harm reduction for substances other than opioids [106, 107], and the increasing adulteration of the unregulated drug supply with fentanyl and xylazine [108–110] are absent, and we urge curriculum developers to collaborate with patients who are actively using drugs to mitigate this lag in relevancy. Demonstration of communication and critical application skills and creation of opportunities for trainees to integrate this knowledge into their practice should be priorities when designing curricula format. We encourage UME and GME programs to publish their harm reduction curricula and hope our iteratively developed Harm Reduction Educational Spectrum can act as a framework for ongoing curricula development, ultimately preparing our physicians-in-training to provide the highest-quality care to people who use drugs.

### Supplementary Information

**Additional file 1.**

## Data Availability

The datasets used and analyzed during the current study are available from the corresponding author on reasonable request.

## Abbreviations

ED: Emergency department
GME: Graduate medical education
HCV: Hepatitis c virus
HIV: Human immunodeficiency virus
MOUD: Medication for opioid use disorder
OSCEs: Observed standardized clinical encounters
OUD: Opioid use disorder
PWID: People who inject drugs
PWUD: People who use drugs
SBIRT: Screening, brief intervention, and referral to treatment
SPs: Standardized patients
SSPs: Syringe services programs
SSTI: Skin and soft tissue infections
SUD: Substance use disorder
UME: Undergraduate medical education
