# Supplementary material for: Harm reduction in undergraduate and graduate medical education: a systematic scoping review
Source: BMC Med Educ. 2023 Dec 21;23:986. doi: 10.1186/s12909-023-04931-9 (PMC10734177; doi:10.1186/s12909-023-04931-9)
Supplement: Supplementary file 1 — Additional file 1. [file 12909_2023_4931_MOESM1_ESM.docx]

Complete Search Strategies

PubMed

731 results on Nov 22, 2021

("harm reduction"[MeSH Terms] OR ("harm"[All Fields] AND "reduction"[All Fields]) OR "harm reduction"[All Fields] OR "naloxone"[MeSH Terms] OR "naloxone"[All Fields] OR "narcan"[All Fields] OR "safer injection*"[All Fields] OR "injection site rotation*"[All Fields] OR "syringe services program*"[All Fields] OR "needle exchange*"[All Fields] OR "syringe exchange*"[All Fields] OR "safe injection site*"[All Fields] OR "supervised injection site*"[All Fields] OR "drug consumption room*"[All Fields] OR "drug consumption facilit*"[All Fields] OR "fentanyl test strip*"[All Fields] OR ("risk reduction"[tiab] AND ("substance use"[tiab] OR "substance abuse"[tiab]))OR ("Risk Reduction Behavior"[Mesh] AND "Substance-Related Disorders"[Mesh]) OR "overdose prevention"[All Fields] OR "prevention of overdose"[All Fields]) AND ("education, medical, undergraduate"[MeSH Terms] OR "undergraduate medical education"[tiab] OR "education, medical, graduate"[MeSH Terms] OR "graduate medical education"[tiab] OR ("education, medical"[MeSH Terms] OR ("education"[tiab] AND "medical"[tiab]) OR "medical education"[tiab]) OR ("medical"[tiab] AND ("curriculum"[MeSH Terms] OR "curriculum"[tiab] OR "curricula"[tiab] OR "curriculums"[tiab] OR "education"[MeSH Subheading] OR "education"[tiab])) OR ("students, medical"[MeSH Terms] OR ("student*"[tiab] AND "medical"[tiab]) OR "medical students"[tiab] OR "medical student"[tiab]) OR "internship and residency"[MeSH Terms] OR "internship*"[tiab] OR "residency"[tiab] OR "residencies"[tiab] OR "residency"[tiab] OR "resident"[tiab] OR "residents"[tiab])

Scopus

497 results Nov 22, 2021

( TITLE-ABS-KEY ( "harm reduction" OR ( "harm" W/1 "reduction" ) OR "naloxone" OR "narcan" OR "safer injection*" OR "injection site rotation*" OR "syringe services program*" OR "needle exchange*" OR "syringe exchange*" OR "safe injection site*" OR "supervised injection site*" OR "drug consumption room*" OR "drug consumption facilit*" OR "fentanyl test strip*" OR ( "risk reduction" AND ( "substance use" OR "substance abuse" ) ) OR ( "Risk Reduction Behavior" AND "Substance-Related Disorders" ) OR "overdose prevention" OR "prevention of overdose" ) AND TITLE-ABS-KEY ( "education, medical, undergraduate" OR "undergraduate medical education" OR "education, medical, graduate" OR "graduate medical education" OR "education, medical" OR ( "education" W/2 "medical" ) OR "medical education" OR ( "medical" W/2 ( "curriculum" OR "curriculum" OR "curricula" OR "curriculums" OR "education" OR "education" ) ) OR "students, medical" OR ( "student*" W/2 "medical" ) OR "medical students" OR "medical student" OR ( ( "medical" OR "clincal" OR "hospital" ) W/2 ( "internship and residency" OR "internship*" OR "residency" OR "residencies" OR "residency" OR "resident" OR "residents" ) ) ) )

ERIC on Ovid

265 results Nov 22, 2021

("harm reduction" or ("harm" and "reduction") or "naloxone" or "narcan" or "safer injection*" or "injection site rotation*" or "syringe services program*" or "needle exchange*" or "syringe exchange*" or "safe injection site*" or "supervised injection site*" or "drug consumption room*" or "drug consumption facilit*" or "fentanyl test strip*" or ("risk reduction" and ("substance use" or "substance abuse")) or ("Risk Reduction Behavior" and "Substance-Related Disorders") or "overdose prevention" or "prevention of overdose").mp.

MedEdPortal

28 results Nov 22, 2021

"harm reduction" OR ("harm" AND "reduction") OR "naloxone" OR "narcan" OR "safer injection*" OR "injection site rotation*" OR "syringe services program*" OR "needle exchange*" OR "syringe exchange*" OR "safe injection site*" OR "supervised injection site*" OR "drug consumption room*" OR "drug consumption facilit*" OR "fentanyl test strip*" OR ("risk reduction" AND ("substance use" OR "substance abuse")) OR ("Risk Reduction Behavior" AND "Substance-Related Disorders") OR "overdose prevention" OR "prevention of overdose"
